# Disparate Utilization of Urine Drug Screen Nationwide in the Evaluation of Acute Chest Pain

**DOI:** 10.5811/westjem.2022.11.58231

**Published:** 2023-03-06

**Authors:** Daniel L. Overbeek, Alexander T. Janke, C.James Watson, Rama A. Salhi, Erin Kim, Dowin Boatright, Eve D. Losman

**Affiliations:** * University of Rochester School of Medicine and Dentistry, Department of Emergency Medicine, Rochester, New York; † Yale School of Medicine, Department of Emergency Medicine, New Haven, Connecticut; ‡ Boston Children’s Hospital, Harvard Medical Toxicology Program, Boston, Massachusetts; § Beth Israel Deaconess Medical Center, Department of Emergency Medicine, Boston, Massachusetts; ¶ Department of Emergency Medicine, New York University Grossman School of Medicine, New York, New York; || University of Michigan, Department of Emergency Medicine, Ann Arbor, Michigan University of Michigan Medical School, Ann Arbor, Michigan; # Institute for Healthcare Policy and Innovation, VA Ann Arbor/University of Michigan, Ann Arbor, Michigan; ** Department of Emergency Medicine, Massachusetts General Hospital, Boston, Massachusetts

## Abstract

**Introduction:**

Urine drug screens (UDS) have unproven clinical utility in emergency department (ED) chest pain presentations. A test with such limited clinical utility may exponentiate biases in care, but little is known about the epidemiology of UDS use for this indication. We hypothesized that UDS utilization varies nationally across race and gender.

**Methods:**

This was a retrospective observational analysis of adult ED visits for chest pain in the 2011–2019 National Hospital Ambulatory Medical Care Survey. We calculated the utilization of UDS across race/ethnicity and gender and then characterized predictors of use via adjusted logistic regression models.

**Results:**

We analyzed 13,567 adult chest pain visits, representative of 85.8 million visits nationally. Use of UDS occurred for 4.6% of visits (95% CI 3.9%–5.4%). White females underwent UDS at 3.3% of visits (95% CI 2.5%–4.2%), and Black females at 4.1% (95% CI 2.9%–5.2%). White males were tested at 5.8% of visits (95% CI 4.4%–7.2%), while Black males were tested at 9.3% of visits (95% CI 6.4%–12.2%). A multivariate logistic regression model including race, gender, and time period shows significantly increased odds of ordering UDS for Black patients (odds ratio [OR] 1.45 (95% CI 1.11–1.90, p = 0.007)) and male patients (OR 2.0 (95% CI 1.55–2.58, p < 0.001) as compared to White patients and female patients.

**Conclusion:**

We identified wide disparities in the utilization of UDS for the evaluation of chest pain. If UDS were used at the rate observed for White women, Black men would undergo nearly 50,000 fewer tests annually. Future research should weigh the potential of the UDS to magnify biases in care against the unproven clinical utility of the test.

## INTRODUCTION

Multiple prior studies have identified racial and gender disparities in emergency department (ED) testing and care. For example, Black patients have been found to be less likely to receive pain medications for acute pain^1^ and less likely to undergo comprehensive evaluations for chest pain.^2^ Gender disparities have also been noted, including in the management of coronary artery disease.^3^ This is further complicated by the possible role of substance use in the development and evaluation of chest pain and coronary artery disease.

Substance use is a critical area in which to consider disparities in acute care, as there are notable societal biases across race and gender that may adversely affect quality and outcomes. These biases have been seen in the opioid epidemic, including inequity in the management of opioid use disorders.^4^ These biases also are entwined with the racialized history of the “War on Drugs” since the 1980s,^5^ including unjustified sentencing practices tied to terminology surrounding the use of powder cocaine and crack cocaine. At the same time, minority communities have been found to be significantly less likely to have treatment facilities available for substance use disorder.^6^

Concern for the possibility of cocaine or stimulant ingestion contributing to a patient’s chief complaint of chest pain is a commonly cited reason for obtaining a urine drug screen (UDS) in the ED.^7^ The UDS tests for metabolites of some common drugs of abuse, including cocaine and amphetamines; however, UDS cannot reliably identify acute intoxication and has a significant false positive rate.^8^ Limited existing empirical work has addressed the usefulness of UDS in the evaluation for acute coronary syndrome, and a positive result on a UDS for cocaine or amphetamine has been found to have no predictive power for the presence of coronary artery disease in patients presenting with chest pain.^7^ When a test has limited clinical utility, disparities in its use should be viewed with increased scrutiny.

### Goals of This Investigation

Our goal was to explore how often UDS is employed in the evaluation of patients presenting with chest pain in a nationally representative sample of ED visits from 2011 to 2019. We hypothesized that UDS utilization would vary significantly across race and gender.

## METHODS

### Design

This was a repeated cross-sectional analysis of the National Hospital Ambulatory Medical Care Survey (NHAMCS) from 2011 to 2019. The NHAMCS is a large dataset of ED visits across the US, which includes demographic data such as race and gender, chief complaint, and UDS use. The NHAMCS data is publicly available from the National Center of Health Statistics, a component of the US Centers for Disease Control and Prevention (CDC). The NHAMCS data is weighted to create a nationally representative dataset, collected via a systematic sampling of a national population of ED visits.^9^

### Sample

The analysis sample was limited to adult ED visits for patients presenting with chief complaints for chest pain or ischemic heart disease. We identified visits regarding chest pain via the “reason for visit” field reported in the NHAMCS, which is coded according to a “Reason for Visit Classification for Ambulatory Care.” The NHAMCS documentation includes the full classification of this coding. Reasons for visit used for inclusion in the study were “chest pain,” “chest discomfort,” “heart pain,” “angina,” and “ischemic heart disease.” Reason for visit was selected over final diagnosis as we considered this to be more closely reflect the ordering practices of clinicians using information available at the time of ordering.

**Population Health Research Capsule**

What do we already know about this issue?

*There is minimal clinical utility of urine drug screens for patients with chest pain. However urine drug screen use may amplify biases in care*.

What was the research question?

*Does ordering of urine drug screens vary for patients presenting with chest pain by race and sex?*

What was the major finding of the study?

*Black male patients had a urine drug screen in 9.3% (95% CI 6.4%–12.2%) of visits for chest pain, compared to 4.6% (CI 3.9%–5.4%) for all patients*.

How does this improve population health?

*Identifying low yield testing that may amplify biases should be a component of interventions targeting health equity*.

### Outcomes and Measures

The primary outcome was whether a UDS was ordered for each visit, which is reported as a binary variable. Rates of UDS ordering were stratified across multiple characteristics, including race, gender, and time trends. Data regarding results of the UDS or specific types of drugs tested was unavailable. In the context of sample size limitations, the race variable was categorized using Black or White racial classification as well as ED visits reporting race as “unknown.”

### Analysis

Survey weights and complex sample design features were implemented to provide nationally representative estimates from the weighted data, and standard errors were adjusted for complex sampling design. We performed analyses in R 4.0.2 (R Foundation for Statistical Computing, Vienna, Austria). All code to reproduce the results are available on request.

## RESULTS

The analysis included 160,526 ED visits (unweighted), including 13,567 chest pain-related visits across nine years, representative of 961 million ED visits (weighted) and 85.8 million ED visits (weighted) for chest pain in that timeframe. Among all ED visits, UDS were ordered for 4.7%. Of the 85.8 million estimated ED visits for chest pain in the study period, for 3.9 million (4.6%) of them a UDS was performed. Table 1 describes the demographics of these ED visits, as well as the subset of visits for chest pain complaints.

**Figure 1:**
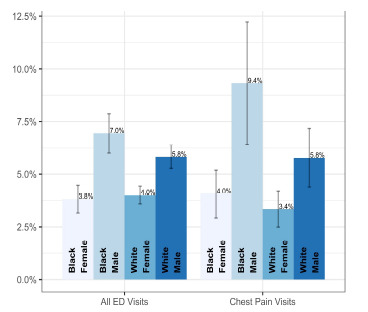
Urine drug screen utilization by gender and race, with 95% confidence intervals.

**Figure 2:**
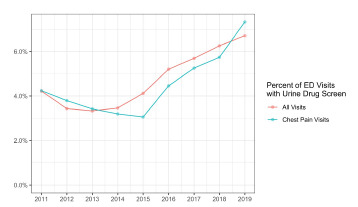
Urine drug screen utilization for all visits and among visits for chest pain by year. *ED*, emergency department.

The rate of UDS utilization in chest pain visits was 4.6% (95% CI 3.9%–5.4%). White females presenting for chest pain had a UDS rate of 3.3% (95% CI 2.5%–4.2%), while Black females had a rate of 4.1% (95% CI 2.9%–5.2%). White males were tested at 5.8% of chest pain visits (95% CI 4.4%–7.2%), and Black males at 9.3% of chest pain visits (95% CI 6.4%–12.2%). Male patients with unknown race were tested at a rate of 5.3% (95% CI 3.0–7.6%), and female patients with unknown race at a rate of 2.5% (95% CI 1.3%–3.6%) (Figure 1). Across the years of the study, UDS utilization was also noted to be increasing. In 2011, chest pain visits had a UDS rate of 4.2%, increasing to 7.3% in 2019. The annual trends are shown in Figure 2.

In a multivariable logistic regression model, including time trends, male gender was associated with increased rates of UDS ordering as compared to female gender (Table 2) (2.00 odds ratio, 95% CI 1.55–2.58). Similarly, Black race was associated with increased odds of UDS ordering as compared to White race (1.45 OR, 95% CI 1.11–1.90).

## DISCUSSION

Despite the lack of clear clinical utility for UDS in the ED evaluation of patients with chest pain, the frequency of UDS testing has grown considerably nationwide and is disproportionately used in the evaluation of Black men with chest pain. Based on the national estimates, if the rate of UDS ordering for Black men were the same as that for White women, Black men presenting to EDs with chest pain would have nearly 50,000 fewer UDS performed per year.

The UDS has poor clinical utility in the ED. In the hospital setting, the drugs tested for vary, but many hospitals perform an immunoassay for metabolites of amphetamines, cocaine, cannabis, opiates, barbiturates, and benzodiazepines. In identifying these metabolites, the urine testing can remain positive for days to weeks after the last use. Additionally, many of the screened drugs have a variety of false positives and false negatives, including common prescribed and over-the-counter medications. In the ED, these characteristics severely limit the ability of the UDS to recognize acute intoxication or identify clinically relevant substance use. Prior work in the toxicology community has argued that due to these issues, the UDS should rarely, if ever, be used to guide management for acute presentations.^8^

Some may argue that there are specific scenarios regarding chest pain presentations where the knowledge of acute cocaine or stimulant intoxication has notable clinical relevance. While the UDS provides information regarding recent exposure, the limitations in acute settings will significantly blunt its ability to guide chest pain workups. Chronic cocaine use has been associated with atherosclerosis; however, existing data has shown no difference regarding the prevalence of coronary artery disease based on a positive UDS in those presenting with chest pain.^7^ Additionally, our results note that the UDS rate for all complaints is similar to those presenting with chest pain (4.7% vs 4.6%, respectively). This further casts doubt on the consideration that UDS be ordered specifically in targeted chest pain evaluations.

Multiple studies have attempted to quantify the prevalence of substance use across populations with conflicting answers. Overall drug use rates are similar across Black and White populations,^10^ with methamphetamine use reported higher in White populations and similar rates of cocaine use in all groups. A recent study shows lower overdose death rates involving methamphetamines in Black populations,^10^ but rates of deaths involving cocaine are higher in Black populations.^12^ Similar rates by gender of positive cocaine or methamphetamine testing have been seen in patients admitted for chest pain observation.^10^ Notably higher rates of methamphetamine use are seen in Native American/Alaskan Native populations;^10^ unfortunately due to the sample size limitations in the NHAMCS, this study could not comment on any ordering disparities regarding that population.

Arbitrary or bias-driven variations within clinical practice are a concern within emergency medicine. Some variation within clinical practice is inevitable, as identical workup and management is not indicated for every presentation for the same chief complaint. However, with increasing awareness of the role of implicit, explicit, and institutional biases, our results underscore the need to consider the utility of the UDS. Further, as drug use continues to be highly stigmatized, consideration must be given to the biased and disparate care that the results of the UDS may create. Given the complicated interplay between healthcare inequities, racism (both structural and interpersonal), and the stigma regarding substance use, it is incumbent upon emergency physicians to recognize how these factors weigh on clinical decision-making. This importance is only magnified when we consider that the clinical utility of the test in question is poorly justified, as in the case of the UDS for chest pain presentations.

## LIMITATIONS

This study has several limitations, primarily related to reliance on a secondary analysis of previously collected data. We did not have a patient-oriented or clinical outcome; future investigations should explore how ordering practices might have downstream consequences for patients. Despite this lack of clinical outcome, there is an absence of empirical data justifying the broad use of UDS in the evaluation of chest pain; and at the same time disparities persist in care access, quality, and outcomes for Black patients. Furthermore, due to sample size limitations, we were unable to address all patient-reported race/ethnicity categories; thus, our study is limited to analyzing only Black and White patients, rather than reflecting the entire emergency care patient population nationally. This inherently does not reflect the complexities of race and ethnicity self-identification, nor can it account for inaccuracies in the collection of this datapoint. However, given the racialized history of drug policy in the US that uniquely targets Black communities, we feel that our results are important despite this limitation.

The NHAMCS data does have some limitations,as with any retrospective data collection, but significant effort is taken by the CDC to maximize its utility as a representative sample.^9^ Additionally, the NHAMCS does not provide the information to analyze hospital-level variation of the disparities identified in this study, which will need to be analyzed with alternative sources of data. Specifically, our study highlights the need to understand whether the increased use of UDS among Black patients reflects clinician, hospital, or even regional variation.

## CONCLUSION

In this study we identify notable disparities in UDS use for ED patients presenting for chest pain, with Black male patients having significantly higher odds of receiving a urine drug screening. Given existing work that UDS is not useful for ruling out clinically significant coronary artery disease, alongside the notable limitations of clinical information provided by the test, the emergency medicine community should apply scrutiny to its ongoing use. Going forward, future investigations should consider the mechanisms behind this ordering disparity, as well as possible downstream clinical and non-clinical impacts.

## Figures and Tables

**Table 1:** Characteristics of emergency department visits for chest pain in the 2011–2019 National Hospital Ambulatory Medical Care Survey (weighted counts, rounded to the nearest thousand).

	All Visits	UDS	Visits for Chest Pain	UDS
Age				
18–29	240,938,000(25.1%)	13,013,000(28.6%)	13,325,000(15.5%)	703,000(17.7%)
30–39	169,990,000(17.7%)	9,877,000(21.7%)	13,036,000(15.2%)	933,000(23.5%)
40–49	147,636,000(15.4%)	8,263,000(18.1%)	15,379,000(17.9%)	899,000(22.7%)
50–64	201,702,000(21.0%)	9,932,000(21.8%)	23,610,000(27.5%)	1,089,000(27.5%)
65+	201,491,000(21.0%)	4,449,000(9.8%)	20,485,000(23.9%)	341,000(8.6%)
Race				
White	578,655,000(60.2%)	27,718,000(60.9%)	51,050,000(59.5%)	2,274,000(57.3%)
Black/African American	195,091,000(20.3%)	9,915,000(21.8%)	18,230,000(21.2%)	1,116,000(28.1%)
Asian	14,244,000(1.5%)	425,000(0.9%)	1,352,000(1.6%)	30,000(0.8%)
Native American/Alaska Native	6,037,000(0.6%)	414,000(0.9%)	472,000(0.5%)	10,000(0.3%)
Native Hawaiian/other Pacific Islander	2,469,000(0.3%)	107,000(0.2%)	248,000(0.3%)	3,000(0.1%)
More than one race reported	2,497,000(0.3%)	84,000(0.2%)	211,000(0.2%)	400(0%)
Unknown	162,763,000(16.9%)	6,872,000(15.1%)	14,274,000(16.6%)	533,000(13.4%)
Gender				
Female	550,823,000(57.3%)	21,121,000(46.4%)	47,776,000(55.7%)	1,579,000(39.8%)
Male	410,933,000(42.7%)	24,412,000(53.6%)	38,060,000(44.3%)	2,387,000(60.2%)
Disposition				
Discharge	769,389,000(80%)	25,400,000(55.8%)	59,113,000(68.9%)	2,472,000(62.3%)
Admit	157,051,000(16.3%)	18,259,000(40.1%)	24,256,000(28.3%)	1,415,000(35.7%)
Transfer	33,585,000(3.5%)	1,755,000(3.9%)	2,325,000(2.7%)	72,000(1.8%)
Died	1,731,000(0.2%)	119,000(0.3%)	141,000(0.2%)	7,000(0.2%)
N (%)	961,757,000(100%)	45,533,000(100%)	85,836,000(100%)	3,966,000(100%)

*UDS*, urine drug screen.

**Table 2:** Associations of urine drug screen use in all ED patients using multivariable logistic regression.

	OR	95% CI	P-value
Gender			
Male	1.998	1.550–2.577	<0.001
Female	(ref)		
Race			
Black/African American	1.453	1.110–1.901	0.007
White	(ref)		
Year (linear trend)	1.104	1.036–1.177	0.002

*OR*, odds ratio; *CI*, confidence interval.
